# Experimental Study on the Influence of Different Loading Weights and Placement Forms on Vacuum Sublimation–Rehydration Thawing of Large Yellow Croaker

**DOI:** 10.3390/foods15030467

**Published:** 2026-01-29

**Authors:** Yuyao Sun, Weidong Wu, Shanshan Chen, Nating Xu, Fangran Liu, Anyuan Xue

**Affiliations:** 1 School of Energy and Power Engineering, University of Shanghai for Science and Technology, Shanghai 200093, China; bj2012_0225@126.com (Y.S.); ntxuuu@163.com (N.X.); liufangran@yeah.net (F.L.); 222210084@st.usst.edu.cn (A.X.); 2 Vehicle Energy and Safety Laboratory, Department of Mechanical Engineering, Ningbo University of Technology, Ningbo 315336, China; chenss@nbut.edu.cn

**Keywords:** vacuum sublimation–rehydration thawing, loading weight, placement form, large yellow croaker, thawing uniformity, thawing efficiency

## Abstract

To optimize vacuum sublimation–rehydration thawing (VSRT) for batch frozen food, large yellow croakers were chosen as research objects to investigate the influence of different loading weights and placement forms on thawing efficiency, uniformity, effect and energy consumption. The results indicated that varying the loading weight significantly influenced the ice crystal sublimation amount per unit mass of large yellow croakers and the water vapor condensation efficiency. For the system in this paper, the optimal loading weight was 1000 g, which resulted in shorter thawing time (96.25 min), the highest thawing uniformity, the optimal thawing effect and lower total specific energy consumption (SEC) of 2.243 MJ/kg. A loading weight of 2000 g was identified as the maximum for rapid thawing, yielding the shortest specific thawing time (67.83 min/kg). Different placement forms altered the water vapor diffusion path, thereby affecting the condensation efficiency. The application of staggered placement form facilitated faster and more even water vapor diffusion, leading to reduced thawing time (91.75 min), improved thawing uniformity, enhanced thawing effect and lower total SEC (2.185 MJ/kg). Compared with other thawing methods, VSRT exhibited superior performance. Additionally, compared to vacuum steam thawing, the total SEC of VSRT was reduced by 76.17~81.53%.

## 1. Introduction

The large yellow croaker (*Larimichthys crocea*) is a highly nutritious and protein-rich fish, which is an economically important species in marine aquaculture, extensively cultivated in regions such as Ningde and Zhoushan, China [1,2,3]. However, because of the high enzyme activity and moisture content, it is susceptible to bacterial contamination, which can lead to fat oxidation, texture deterioration and protein deterioration, ultimately diminishing the commercial value [4,5]. To address these issues, low-temperature frozen storage is commonly adopted to preserve the quality of fish for extended periods [6]. Thawing is an essential procedure before further processing frozen food, as it significantly influences the protein, moisture status and muscle tissue structure of fish [7,8]. Therefore, identifying a suitable thawing method is critical to ensure both effective quality preservation in thawed large yellow croaker and high thawing efficiency [9].

Vacuum steam thawing (VST) utilizes the latent heat released from water vapor condensation in a low-pressure, low-oxygen environment [10]. Compared to both air thawing (AT) [11] and water thawing [12], VST can obtain shorter thawing time, greater antibacterial property and enhanced antioxidant capacity, thereby better preserving the quality of food after thawing [13,14]. Other relatively efficient thawing methods, such as ultrasonic thawing [15] and microwave thawing [16], achieve rapid thawing through cavitation effects and polar molecular vibrations, respectively. However, the attenuation of ultrasonic waves during propagation and the limited penetration depth of microwaves can result in localized overheating, which can diminish the quality of food [17]. In contrast, VST could achieve more uniform heat distribution, which improves thawing uniformity and food quality preservation. To optimize this VST process, our research group has proposed vacuum sublimation–rehydration thawing (VSRT). During VSRT, the ice crystal sublimation creates numerous tiny pores within frozen food. These tiny pores promote condensation heat exchange among water vapors and frozen food [18]. This mechanism enhances the thawing efficiency and improves the quality of thawed food. Kopec et al. [19] compared the performance of VSRT and VST in thawing pork and demonstrated that the former achieved higher thawing efficiency and superior quality in the thawed pork.

Current research on VSRT primarily focuses on optimizing the processing for single-item frozen food, whereas studies on batch processing remain limited. However, the food industry increasingly operates in large-scale processing scenarios, where single-item thawing modes are insufficient to meet demands for thawing efficiency and energy consumption [20]. Therefore, conducting in-depth research on batch thawing holds greater application value. Zhang [21] focused on the batch thawing process and developed an “efficient batch unfreezing system for frozen food”, which utilized flexible thermal water vapor to achieve uniform thawing of batch frozen food. Wei et al. [22] investigated the placement form of frozen food and designed a “cryopreservation device capable of unfreezing in batches”, which could achieve uniform thawing of multiple frozen foods using a modular cylindrical structure. To systematically evaluate the performance of VSRT in batch thawing, large yellow croakers with initial temperatures of −18 °C (each weighing approximately 250 g) were selected as research objects. The influence of different loading weights and placement forms on thawing efficiency, uniformity, effect and energy consumption was studied. Moreover, VSRT was compared with other thawing methods (AT, VST). The aim was to optimize this thawing technology and provide both reference and guidance for applying VSRT in the industrial-scale processing of batch frozen food.

## 2. System Fundamentals and Components

The VSRT process comprises a sublimation stage and a rehydration stage [23]. During the sublimation stage, once the pressure in the vacuum chamber (P_VC_) drops below that saturation pressure of ice crystals within frozen food (the saturation pressure of ice crystals at −18 °C was 125 Pa [24]), ice crystal sublimation occurs. Then, the ice crystal sublimation becomes water vapor and escapes from frozen food, leaving behind numerous tiny pores [25]. At this stage, the pressure in frozen food reaches equilibrium with the P_VC_, both maintained at a low pressure by a vacuum pump unit. In the rehydration stage, after turning on rehydration valves, the saturated water vapor produced by rehydration equipment is injected into the vacuum chamber, which leads to a rapid pressure increase in the vacuum chamber. However, the tiny pores within frozen food are small and numerous, which can hinder the rapid permeation and the quick equilibrium attainment of water vapor [26]. Thus, a pressure difference between the P_VC_ and the internal pressure of frozen food is created. Due to this pressure difference, the water vapor spreads to the internal tiny pores and the surface of frozen food, where it condenses and releases latent heat. This process facilitates the heat transfer in frozen food, and replenishes the moisture lost during ice crystal sublimation [27].

**Figure 1:**
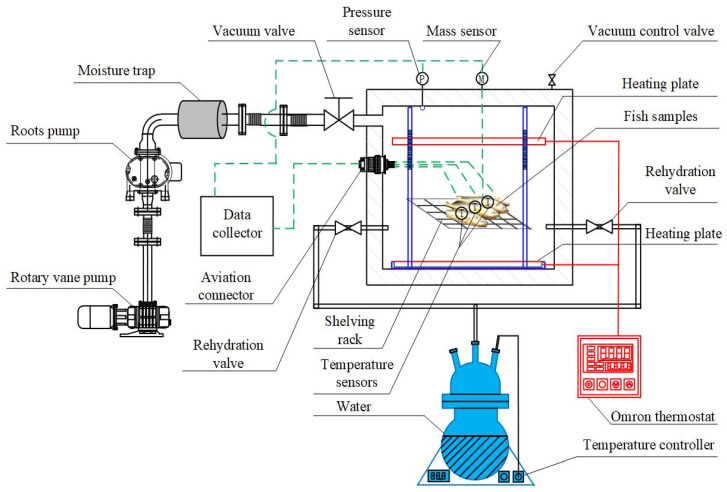
VSRT system.

As illustrated in Figure 1, the VSRT system is primarily composed of a vacuum chamber (internal dimensions of 600 mm (L) × 460 mm (W) × 400 mm (H)), data acquisition equipment, heating equipment, vacuum equipment and rehydration equipment. The vacuum equipment includes the vacuum pump unit (rotary vane pump and Roots pump), a moisture trap, a vacuum control valve and a vacuum valve. Through the coordinated operation of the vacuum pump unit and the vacuum valve, the P_VC_ can keep within a specific range. Additionally, the moisture trap absorbs residual vapor to prevent it from entering the vacuum pump unit, thereby protecting the performance of pumps. The heating equipment consists of two heating plates (symmetrically double-sided heating, 420 mm × 400 mm), a shelving rack (420 mm × 400 mm) and an Omron thermostat. The heating plate temperature is controlled by the Omron thermostat (the temperatures at all parts of heating plates are consistent). The heating-plate-to-frozen-food distances (D_H-F_) of two heating plates are the same. The shelving rack is utilized to place frozen food. The heating plates fully cover the entire shelving rack. The rehydration equipment comprises two rehydration valves and a water vapor generator (equipped with a temperature controller). This equipment generates sufficient saturated water vapor, whose temperature is controlled by a temperature controller. The data acquisition equipment is equipped with a mass sensor, multiple temperature sensors (T-type thermocouples), a pressure sensor, a data collector and an aviation connector. This equipment can real-time monitor and record data, such as the P_VC_, the core temperature of frozen food, and the mass of frozen food. The aviation connector is utilized to link signal lines. The equipment and instruments employed in this study are listed in Table 1. Prior to all experiments, pressure sensors, temperature sensors, mass sensors, and other instruments were calibrated according to the specifications provided in Reference [28]. The resulting accuracies are summarized in Table 1.

## 3. Experimental Procedures and Data Processing

### 3.1. Experimental Procedures

#### 3.1.1. Preparation of Samples

**Figure 2:**
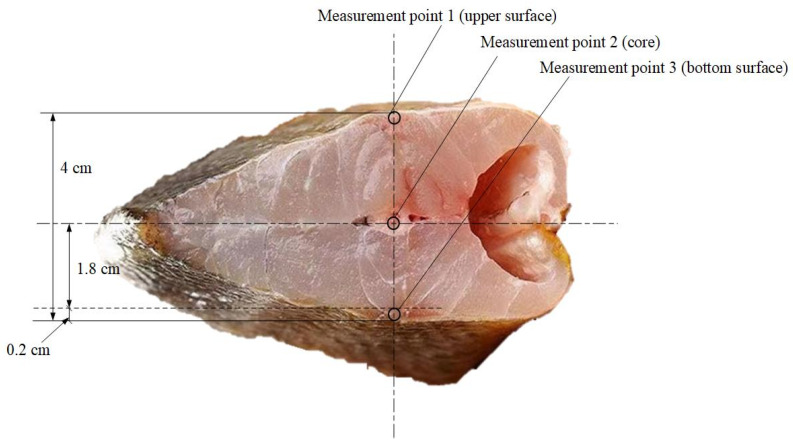
Cross-sectional view of thermocouple measurement points in a large yellow croaker.

(1) The fresh large yellow croaker samples (250 ± 3 g) were bought from Shanghai Luchao Port (Shanghai, China). They were stored in a large amount of ice cubes, and transported to the laboratory within 1 h.
(2) Random samples of large yellow croakers with uniform specifications were selected for the experiments. The initial parameters of chosen fish samples were as follows: mass of 250 ± 3 g, brightness of 77.20 ± 0.11, redness of 4.49 ± 0.09, yellowness of 26.85 ± 0.10, pH of 6.51 ± 0.01, hardness of 2416.8 ± 151.2 gram-force (gf), springiness of 0.73 ± 0.02, adhesiveness of 6.84 ± 0.38 g·s and cohesion of 0.36 ± 0.03.
(3) As shown in Figure 2, thermocouples (with a diameter of 0.2 cm) were inserted at designated measurement points in fish samples, which were used to measure the upper surface temperature (insertion depth of 0.2 cm), core temperature (insertion depth of 2 cm) and bottom surface temperature (insertion depth of 0.2 cm) of fish samples.
(4) According to the standards of SC/T3101-2010 [29] and NYT3524-2019 [30], the core temperatures of fish samples were set at −18 °C as the initial thawing temperature. The target thawing temperature was defined as 4 °C (the fastest-thawed fish sample), while the core temperatures of remaining fish samples were maintained between 0 °C and 4 °C.
(5) All fish samples were first frozen in a −40 °C freezer for 10 h until their core temperatures reached −18 °C [31]. Following this, they were transferred to a −18 °C freezer for 10 h storage. After completing the above operations, take fish samples from the freezer for experiments. The freezers used were air-cooled freezers (Model: BD/BC-100KEMS) produced by Midea Group Co., Ltd. (Foshan, China), with a minimum cooling temperature of −40 °C and a maximum freezer load of 100 L.

#### 3.1.2. Experiment of VSRT

After powering on heating plates and waiting for their temperatures to stabilize, the fish samples were positioned on the shelving rack. Then, after sealing the vacuum chamber door, the vacuum pump unit was operated. When the P_VC_ declined under 610 Pa, the sublimation stage began. The P_VC_ was maintained at about 30 Pa for a long time during this stage. Upon reaching the preset sublimation time, the vacuum valve was turned off, and the rehydration valves were subsequently turned on, which represented the start of the rehydration stage. Since the P_VC_ was below the saturated pressure of water (at a set rehydration temperature) in the water vapor generator, the water rapidly boiled and generated saturated water vapor. The water vapor could be introduced into the vacuum chamber due to a pressure difference to condense with fish samples. After the core temperatures of fish samples reached the target thawing temperatures, the mass change, color difference, pH and other parameters were measured. These parameters were compared with those of fresh samples.

**Figure 3:**
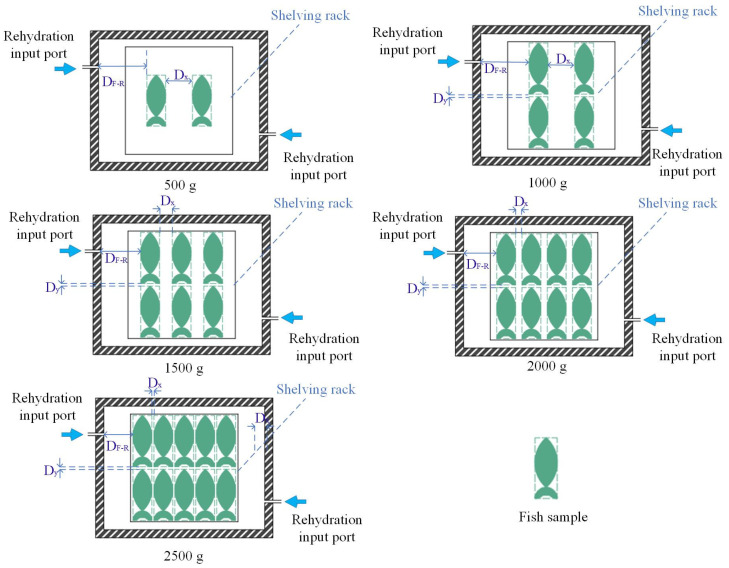
Placement forms of large yellow croakers with different loading weights (overhead cross-sectional view); D_x_ is the lateral distance between adjacent fish samples; D_y_ is the longitudinal distance between adjacent fish samples; D_F-R_ is the shortest distance from fish sample to rehydration input port.

Based on single-factor experiments and full-factor experiments of VSRT [28], the optimal condition for thawing large yellow croakers was obtained, which was as follows: sublimation time of 20 min, D_H-F_ of 80 mm and rehydration temperature of 35 °C, and heating plate temperature of 35 °C. The initial environmental temperature was set at 15 °C. To further explore the optimal loading weight based on the optimal operation condition, the loading weights were set to 500, 1000, 1500, 2000, and 2500 g, using a uniform placement form (as shown in Figure 3 and Table 2). The batch fish sample was to consist of no less than two fish (500 g). Ten fish samples (2500 g) represented the maximum number that could be loaded on a shelving rack in this VSRT system. Therefore, a loading weight range of 500 g to 2500 g was selected for this study. Additionally, the loading weight could be converted into the loading ratio, so the study results could be generalized to VSRT systems of different volumes. Moreover, based on the influence of different loading weights on VSRT, the optimal loading weight was determined and subsequently applied in comparative experiments involving different placement forms. To reduce the effect of individual variability among fish samples on the experimental data, each experiment was repeated three times. Each evaluation index was measured three times, and the average value was taken.

#### 3.1.3. Experiment of AT

The fish samples were placed on a plate without any heat source and thawed by ambient air (15 ± 1 °C) [28]. Once the core temperature of fish samples reached the target thawing temperature, parameters such as mass changes, color differences, and pH value were measured, and were compared to those of the fresh sample.

#### 3.1.4. Experiment of VST

The fish samples were put on the shelving rack, with heating plates off. After sealing the vacuum chamber door, the P_VC_ was stabilized by vacuum equipment (5 ± 0.1 kPa) [32]. Simultaneously, the water vapor generator was switched on, continuously supplying saturated water vapor at 35 °C to condense with fish samples. Once the core temperature of fish samples reached the target thawing temperature, parameters such as mass changes, color differences, and pH value were measured. These parameters were compared to those of the fresh sample.

### 3.2. Evaluation Indexes

#### 3.2.1. Thawing Efficiency (Thawing Time, Thawing Rate, Specific Thawing Time)

Thawing efficiency is commonly evaluated by thawing rate, thawing time and specific thawing time (*STT*) in the batch thawing of frozen food. Thawing time is defined as the period from the start of thawing until the core temperature of frozen food reaches the target thawing temperature. The thawing rate can quantify the influence of the geometric characteristics on frozen food during the thawing process [33,34], which is calculated using Formula (1). The *STT* represents the time required for thawing per unit mass of frozen food [35], which is calculated using Formula (2).

(1) $$v=\frac{D}{t_{1}}$$

(2) $$STT=\frac{t_{thaw}}{M_{0}}$$

where *D* represents the shortest distance between the surface and the core of the fish sample, cm; *v* represents the thawing rate, cm/h; STT represents the specific thawing time, min/kg; $t_{1}$ represents the duration from the surface temperature reaches 0 °C until the core temperature reaches 4 °C, h; $M_{0}$ represents the mass of fish sample before thawing, kg; and $t_{thaw}$ represents thawing time, min.

#### 3.2.2. Thawing Uniformity

Thawing uniformity describes the temperature dispersion among measurement points after thawing, which can be quantified by temperature coefficient of variation (*TCOV*) [36]. This parameter indicates the average percentage of relative fluctuation of the temperature at each measurement point compared to the average temperature value. Lower *TCOV* indicates greater thawing uniformity, and it is calculated by Formula (3):

(3) $$TCOV\;=\;\frac{1}{T_{ave-t}}\sqrt{\frac{1}{n-1}\sum_{i=1}^{n}{\left(T_{i-t}-T_{ave-t}\right)}^{2}}\times 100\%$$

where $T_{ave-t}$ represents the average temperature among all measurement points, °C; TCOV represents temperature coefficient of variation; $T_{i-t}$ represents the temperature of each measurement point, °C; and n is the number of measurement points.

#### 3.2.3. Thawing Loss

Thawing loss refers to the percentage of mass loss in food after thawing [37], which is used to quantify the degree of juice loss. It can be calculated by Formula (4) (taking the average value of each fish sample):

(4) $$\eta =\frac{\left.M_{0}-M_{1}\right.}{M_{0}}\times 100\%$$

where *η* represents thawing loss; *M*_1_ represents the fish sample mass after thawing, kg.

#### 3.2.4. Moisture Content

Moisture content refers to the proportion of moisture contained within food. According to the GB 5009.3-2016 standard [38], a 10 g fish sample was weighed and placed on the shelving rack in the vacuum chamber for vacuum drying. During the process, the P_VC_ was maintained at 40~53 kPa, and the heating plate temperature was controlled at 60 ± 5 °C. The drying continued until the mass sensor recorded a constant mass, indicating that the sample was completely dry. The moisture content of fish sample was then calculated by Formula (5) (taking the average value of each fish sample):

(5) $$\omega =\frac{\left.M_{0}-M_{d}\right.}{M_{0}}\times 100\%$$

where ω represents moisture content; *M*_d_ represents the fish sample mass after drying, kg.

#### 3.2.5. Color Difference

Color difference can reflect the freshness of food after thawing. Brightness, redness and yellowness are measured by colorimeter. The color difference is calculated by Formula (6) (taking the average value of each fish sample) [39]:

(6) $$∆E=\sqrt{{\left(a^{*}-a^{**}\right)}^{2}+{\left(b^{*}-b^{**}\right)}^{2}+{\left(L^{*}-L^{**}\right)}^{2}}$$

where ∆E represents color difference; b represents yellowness; L represents lightness; a represents redness; ** indicates thawing completed; * indicates before thawing.

#### 3.2.6. pH Parameter

The pH can assess the degree of acidity or alkalinity of food after thawing. If the pH value of the thawed fish sample is closer to that of fresh fish, the freshness of this fish sample will be higher. The measurement was performed according to the following method [40]. Firstly, the fish sample (10 g) was ground using a meat grinder (brand: Royalstar; model: QC, Hefei Royalstar Electronic Appliance Group Co., Ltd., Hefei, China). Next, deionized water (100 mL) was added. In addition, the mixture was shaken to be homogeneous. Then, following a 30 min standing period, the mixture was filtered by filter paper. Finally, the filtrate pH was determined with a pH meter (taking the average value of each fish sample). The pH meter was calibrated using the three-point calibration method in the constant temperature laboratory. Before calibration, the chamber was supplied with 15 °C constant-temperature air for 6 h to stabilize the ambient temperature at 15 °C, using an air handling unit. The calibration processes of the pH meter were as follows: (1) First, after activating the pH meter, its electrode was immersed in a pH-4.00 standard buffer solution. Once the pH measured parameter stabilized and the pH meter indicated successful calibration, the electrode was removed and wiped clean. (2) Next, the electrode of pH meter was immersed in a pH-6.90 standard buffer solution. Once the pH measured parameter stabilized and the pH meter indicated successful calibration, the electrode was removed and wiped clean. (3) Finally, the electrode of the pH meter was immersed in a pH-9.28 standard buffer solution. Once the pH measured parameter stabilized and the pH meter indicated successful calibration, the calibration was complete. Otherwise, the electrode was cleaned again and the above steps were repeated until the pH meter showed successful calibration.

#### 3.2.7. Texture Parameters

The texture parameters comprise hardness, cohesiveness, adhesiveness and springiness, which characterize the textural and sensory properties of food after thawing. Hardness is the maximum force required to achieve a deformation of the sample, and its value equals the peak force of the first compression. Springiness refers to the ability of a sample to return to its original height or shape after the first deformation upon removal of the external force. It equals the ratio of the time required for the second compression to reach the target deformation to the time required for the first compression to reach the target deformation. Adhesiveness is the power required to separate the probe from the surface of the sample, and its value equals the area of negative peak generated during the withdrawal of the probe after the first compression. Cohesiveness is the strength of the internal structure of the sample to resist breakage and maintain integrity, and its value equals the ratio of the area under the positive force region of the second compression curve to that of the first compression curve. The measurement processes were as follows. Fish samples were trimmed into cuboids (20 mm (L) × 20 mm (W) × 10 mm (H)). The texture analysis was conducted on a TA-XT2i texture analyzer using a P/50 probe (taking the average value of each fish sample). We used the following test parameters: compression distance of 10 mm, trigger force of 5 g, pre-measurement rate of 3.00 mm/s, in-measurement rate of 1.00 mm/s, post-measurement rate of 1.00 mm/s, and compression interval of 5 s.

#### 3.2.8. Specific Energy Consumption (SEC) Parameter

The SEC serves as a critical metric for evaluating energy utilization efficiency. The total SEC comprises the SEC of vacuum equipment (SEC-VE), the SEC of heating equipment (SEC-HE, which includes preheating), and the SEC of the rehydration equipment (SEC-RE). The SEC is calculated by Formula (7) [41]:

(7) $$\psi =\frac{E_{i}}{M_{0}}$$

where ψ represents specific energy consumption, MJ/kg; $E_{i}$ represents energy consumption, MJ.

### 3.3. Normalization Evaluation

The normalization evaluation is used to clearly represent the performance of evaluation indexes under different conditions, with 1.00 indicating the best and 0 indicating the worst. For parameters where larger values mean better (such as brightness), their scores can be obtained using Formula (8). For parameters where smaller values mean better (such as *STT*), their scores can be obtained using Formula (9) [42].

(8) $$Y_{S}=\frac{Y-Y_{min}}{Y_{max}-Y_{min}}$$

(9) $$Y_{S}=1-\frac{Y-Y_{min}}{Y_{max}-Y_{min}}$$

where $Y_{S}$ represents evaluation index score; Y represents value of evaluation index; $Y_{max}$ represents the maximum evaluation index value; and $Y_{min}$ represents the minimum evaluation index value.

### 3.4. Uncertainty Analysis and Data Statistics

The absolute uncertainties of directly measured parameters (such as temperature and pressure) can be calculated by Equation (10), and the combined uncertainties of calculated parameters (such as thawing rate and *STT*) are calculated by Equations (11) and (12) [43]. In this study, evaluation indexes comprise thawing rate, *STT*, *TCOV*, SEC, color difference and thawing loss. Based on the calculation, their uncertainties are 0.12%, 0.20%, 0.28%, 0.06%, 0.23%, and 0.14%, respectively. The data in this paper are processed using SPSS 22.0 software. The significances differences in this paper are compared based on Duncan’s multiple-range test (*p* < 0.05).

(10) $$u=\frac{a}{\sqrt{3}}$$

(11) $$Y=f(x_{1},x_{2},\;\cdots x_{j})$$

(12) $$u_{Y}={\left[\sum_{i=1}^{N}{\left(\frac{\partial Y}{\partial x_{i}}\right)}^{2}u^{2}\left(x_{i}\right)\right]}^{\frac{1}{2}}$$

where u represents absolute uncertainty; a represents accuracy; $f(x_{1},x_{2},\cdots x_{j})$ represents calculation function; $u_{Y}$ represents combined uncertainty; and $x_{i}$ represents input variate.

## 4. Results and Discussion

### 4.1. Influence of Different Loading Weights on VSRT

#### 4.1.1. Thawing Process

**Figure 4:**
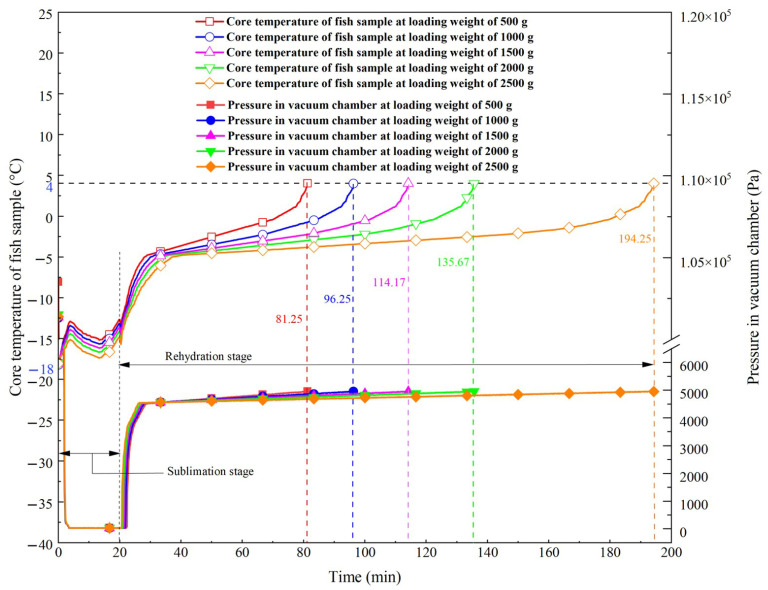
Thawing curves of VSRT under different loading weight conditions.

During the VSRT process, both the P_VC_ and the core temperature of fish samples vary with loading weights. The experiments of thawing were conducted on the basis of the method described in Section 3.1.2. The thawing curves under different loading weight conditions are presented in Figure 4, and the total masses of fish samples at different stages are presented in Table 3.

As shown in Figure 4, during the sublimation stage, the P_VC_ dropped quickly and stabilized at approximately 30 Pa. During the rehydration stage, the P_VC_ rose rapidly and then continued to increase slowly until thawing was completed. This phenomenon occurred because the vacuum pump unit operated continuously and efficiently during the sublimation stage, which caused the P_VC_ to fall below the saturation pressure required for ice crystal sublimation from fish samples. Thus, the sublimation of ice crystals was facilitated. During the rehydration stage, once the rehydration valves were turned on, the saturated water vapor (35 °C) was continuously injected into the vacuum chamber, leading to a rapid increase in P_VC_. As the injection rate and condensation rate of water vapor reached equilibrium, the increasing rate of P_VC_ slowed gradually and stabilized. As the loading weight increased, the P_VC_ started a slow upward process more quickly (the time of this process was Time-1), but required a longer time to stabilize (the time of this process was Time-2). In this stage, when the loading weights were 500, 1000, 1500, 2000 and 2500 g, Time-1 was 8.58, 7.75, 7.50, 6.92 and 6.25 min, while Time-2 was 52.67, 68.50, 86.67, 108.75 and 168.00 min. This phenomenon was attributed to the reduced volumetric gas phase space (VGPS) in vacuum chamber as the loading weight increased. According to the Clausius–Clapeyron equation, a smaller VGPS resulted in a higher initial rate of pressure increase [26]. However, the increased number of fish samples introduced more “pathways between adjacent fish samples” (PW_F-F_) for water vapor to diffuse, and expanded the surface area available for condensation heat exchange, which increased the difficulty of pressure stabilization. Therefore, the time required for the P_VC_ to reach a stable state was prolonged.

According to the core temperature curves in Figure 4, during the sublimation stage, the temperature first rose, then declined, and finally enhanced again. During the rehydration stage, it first rose rapidly to −5 °C, then increased slowly to −1 °C, and finally rose rapidly again to 4 °C. The mechanisms underlying these phenomena were explained as follows. During the sublimation stage, the sudden drop of P_VC_ caused the rapid freezing of incompletely frozen collagens and myofibrillar proteins in the fish samples, which released latent heat, thus leading to a short-term rapid increase in the core temperature of the fish samples. Afterwards, as a lot of ice crystals sublimated into water vapor by absorbing heat, the core temperature of fish samples decreased. However, the continuous heating supplied by heating plates provided compensatory thermal energy, causing the core temperature of the fish samples to eventually rise again. With constant sublimation time and heating plate temperature, an increase in loading weight led to fewer ice crystals sublimated per fish sample and less radiant heat absorbed from the heating plates, which thereby reduced the peak temperature rise in the core temperature of the fish samples in the sublimation stage. In the rehydration stage, the initial fast temperature enhancement was due to the release of latent heat from water vapor condensing within the tiny pores and on the surface of the fish samples. The maximum ice crystal melting zone (MICMZ) of large yellow croakers was −5~−1 °C [44]. Within this range, the significant heat demand for ice crystal melting led to a slower rate of temperature increase. After breaking through the MICMZ, the heat required was primarily sensible heat, causing the temperature to rise more rapidly until thawing was completed.

As presented in Table 3, the total mass of fish samples decreased in the sublimation stage because of the ice crystal sublimation, while it increased again during the rehydration stage due to the moisture replenishment via water vapor condensation. In the sublimation stage, higher loading weight increased the total ice crystal content in fish samples, and ice crystal sublimation occurred simultaneously in each fish sample. Thus, the total ice crystal sublimation amount enhanced with increasing loading weight. However, the increased loading weight also reduced the average radiant heat for per fish sample to promote ice crystal sublimation, which consequently lowered the ice crystal sublimation ratio. At this stage, when the loading weight was 500 g, the ice crystal sublimation amount was the smallest (27.08 g), but the ice crystal sublimation ratio was the highest (5.42%). In contrast, when the loading weight was 2500 g, the ice crystal sublimation amount was the largest (64.50 g), but the ice crystal sublimation ratio was the lowest (2.58%). In the rehydration stage, a higher ice crystal sublimation ratio facilitated the formation of more tiny pores, which enhanced moisture replenishment through vapor condensation. Additionally, an appropriate width of PW_F-F_ was conducive to the efficient diffusion of water vapor around fish samples, leading to more efficient condensation and rehydration. At a loading weight of 1000 g, both the ice crystal sublimation ratio and the width of PW_F-F_ were optimal, which resulted in the highest water replenishment ratio (3.81%). The highest water replenishment ratio indicated that the water vapor condensation efficiency was the highest.

#### 4.1.2. Thawing Efficiency and Thawing Uniformity

**Figure 5:**
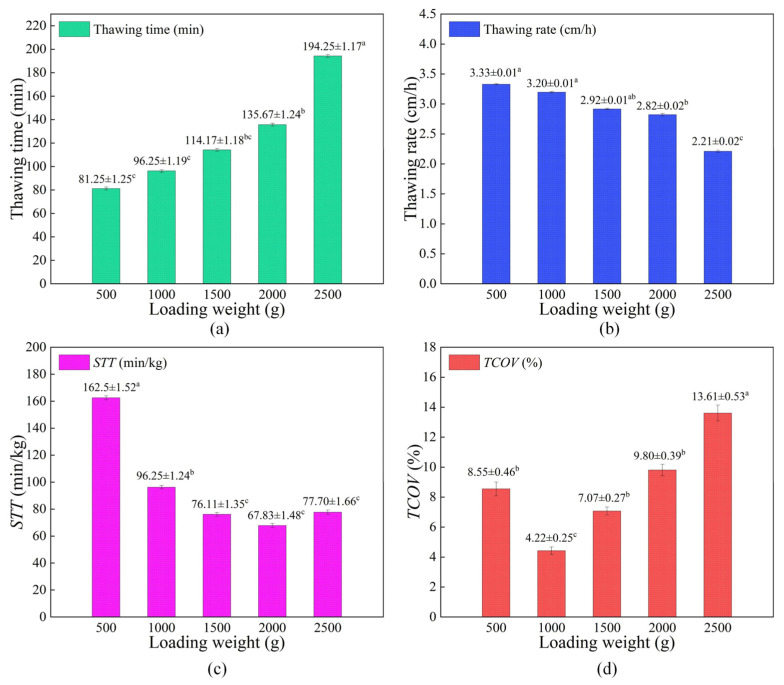
Changes in thawing efficiency and thawing uniformity with various loading weight conditions; (**a**) thawing time; (**b**) thawing rate; (**c**) STT; (**d**) TCOV; within the same subfigure, different superscripted letters indicate significant differences (*p* < 0.05).

Batch thawing aims to achieve both high thawing efficiency and high thawing uniformity, thereby maintaining the superior quality of food after thawing. The changes in thawing rate, thawing time, *STT* and *TCOV* with various loading weight conditions are shown in Figure 5.

As illustrated in Figure 5a and Figure 5b, when the loading weights were 500, 1000, 1500, 2000, and 2500 g, the thawing times were 81.25, 96.25, 114.17, 135.67, and 194.25 min, while the thawing rates were 3.33, 3.20, 2.92, 2.82 and 2.21 cm/h, respectively. With increasing loading weight, the thawing time rose, while the thawing rate declined. The primary reasons for these trends were as follows. Firstly, larger loading weight increased the total ice crystal amount (i.e., the thawing load), thereby increasing the thermal energy required to melt ice crystals during thawing. On the other hand, larger ice sublimation amount contributed to enhancing water vapor condensation efficiency, which accelerated ice melting and improved thawing efficiency. However, within the loading weight range of 500 g to 1000 g, the extent of improvement in thawing efficiency was significantly less pronounced than the increase in thermal demand. As a result, increasing the loading weight could reduce the thawing rate and prolong the thawing time.

As presented in Figure 5c, for loading weights of 500, 1000, 1500, 2000, and 2500 g, the *STT* values were 162.5, 96.25, 76.11, 67.83, and 77.70 min/kg, respectively. When the loading weight rose, the *STT* decreased initially and then increased, reaching a minimum value of 67.83 min/kg at a loading weight of 2000 g. This trend occurred because the total ice crystal sublimation increased with enhanced loading weight, which could promote the formation of more tiny pores. Numerous tiny pores could improve condensation heat exchange between water vapor and fish samples. However, when the loading weight exceeded 2000 g, the increment in ice crystal sublimation decreased significantly, leading to a reduced increment of tiny pore formation. Consequently, the heat released from water vapor condensation was insufficient to support rapid ice melting, which prolonged thawing time and caused *STT* to increase.

As shown in Figure 5d, for loading weights of 500, 1000, 1500, 2000, and 2500 g, the *TCOV* values were 8.55%, 4.22%, 7.07%, 9.80%, and 13.61%, respectively. The *TCOV* first decreased and then increased with enhanced loading weight, reaching its lowest value (4.22%) at 1000 g. This phenomenon was because at lower loading weight (500 g), the excessively wide PW_F-F_s resulted in diffusion hysteresis, which was not conducive to uniform water vapor distribution. Conversely, at higher loading weights (≥1500 g), overly narrow PW_F-F_s restricted the diffusion of water vapor, which caused the water vapor to preferentially condense with fish samples near the rehydration input ports. Thereby, the temperature differences among fish samples at different locations were amplified. In comparison, at 1000 g loading weight, the vapor distribution was optimal under the operation condition, facilitating highly efficient and uniform condensation heat exchange.

#### 4.1.3. Thawing Loss and Moisture Content

Thawing loss is a direct indicator of juice loss in fish samples after thawing. A lower thawing loss indicates better water retentivity in large yellow croakers, which is associated with higher market value [45]. This can also be reflected in the relatively higher moisture content of fish samples. The variation in thawing loss and moisture content under different loading weight conditions is shown in Table 4.

As presented in Table 4, when the loading weights were 500, 1000, 1500, 2000, and 2500 g, the thawing losses were 1.67%, 1.24%, 1.40%, 1.56%, and 2.12%, and the moisture content were 72.90%, 73.33%, 73.17%, 73.01% and 72.44%, respectively. With increasing loading weight, the thawing loss first decreased and then enhanced, reaching the minimal value (1.24%) at a loading weight of 1000 g. At the same time, the moisture content of fish samples also reached the highest value (73.33%). This trend was attributed to the combined effects of thawing uniformity and thawing time, with the former being the dominant factor [46]. At a loading weight of 1000 g, the width of PW_F-F_ was appropriate, which promoted the rapid and uniform diffusion of water vapor. This diffusion characteristic favored efficient and even condensation heat exchange, which prevented the formation of sharp-shaped ice crystals and avoided cell membranes being punctured, thus reducing juice loss. Moreover, under this loading weight condition, shorter thawing time reduced the period of environmental exposure for fish samples. This was beneficial to minimize both juice dripping loss and dry loss, thereby better enabling fish sample to maintain a higher moisture content. Therefore, under the given operation condition, the lowest thawing loss could be obtained at a loading weight of 1000 g.

#### 4.1.4. Color

The colors of fish samples can directly reflect their freshness, mainly represented by brightness, redness, yellowness and color difference (Δ*E*). Fish samples with smaller Δ*E* are associated with higher market values [47]. The variation in color under different loading weight conditions is presented in Table 4.

As presented in Table 4, for loading weights of 500, 1000, 1500, 2000, and 2500 g, the lightness values of fish samples were 76.83, 77.08, 76.95, 76.77 and 75.43; the redness values were 3.82, 3.99, 3.91, 3.84 and 3.65; the yellowness values were 31.13, 30.78, 30.92, 31.08 and 32.33; and the Δ*E* values were 4.35, 3.97, 4.12, 4.30 and 5.82, respectively. Compared with the fresh sample, as the loading weight increased, the change in brightness (Δ*L*), change in redness (Δ*a*), and change in yellowness (Δ*b*) all decreased initially and then increased, while the Δ*E* exhibited a trend of decreasing initially and then increasing. When the loading weight was 1000 g, the Δ*L*, Δ*a* and Δ*b* were the smallest, and the Δ*E* was the lowest (3.97). The reasons for these variations were as follows. First, this result was attributed to high water vapor condensation efficiency and appropriate width of PW_F-F_, meaning that ice crystals melted rapidly and uniformly, which reduced cellular damage and thereby minimized juice loss. Moreover, the rapid and uniform thawing decreased the contact among proteins, lipids and oxidases within fish samples, thus reducing the extent of denaturation and oxidation [48]. At a loading weight of 1000 g, the condensation efficiency was the highest and the width of PW_F-F_ was the optimal, which minimized the lipid oxidation, juice loss and protein denaturation to the greatest extent. Consequently, the Δ*L*, Δ*a* and Δ*b* of fish samples after thawing were all the smallest at this loading weight condition, and the lowest Δ*E* was achieved.

#### 4.1.5. pH

The pH serves as a reliable indicator for evaluating the freshness of food after thawing. Compared with the fresh sample, smaller pH change (ΔpH) means higher freshness [49]. The pH of fresh large yellow croakers was measured as 6.51. The change in pH with various loading weight conditions is presented in Table 4.

As shown in Table 4, when the loading weights were 500, 1000, 1500, 2000, and 2500 g, the pH values were 6.56, 6.53, 6.55, 6.59, and 6.63, respectively. The pH decreased initially and then increased with increasing loading weight, reaching the lowest value (6.53) at a loading weight of 1000 g, where the ΔpH was the smallest (+0.02). This trend could be explained by the influence of both thawing time and thawing uniformity on pH. Longer exposure to the thawing environment accelerated the decomposition of nitrogen-containing compounds within fish samples, which led to a more significant increase in the pH value. In addition, uneven thawing caused localized accumulation of alkaline substances in fish samples. These two factors collectively contributed to an increase in pH, with thawing uniformity exerting a better impact on the fish sample quality after batch thawing [20]. Thus, at a loading weight of 1000 g, the ΔpH was minimized.

#### 4.1.6. Texture Parameter

The texture parameters, including adhesiveness, springiness, hardness and cohesiveness, can objectively quantify the textural characteristics and sensory properties of food after thawing. Compared with the fresh sample, minimal changes in these parameters can indicate better preservation of texture and taste. The variation in texture parameters under different loading weight conditions is presented in Table 5.

As shown in Table 5, as the loading weight increased, the hardness, springiness and cohesiveness of the fish sample first increased and then declined, while the adhesiveness first declined and then increased. When the loading weight was 1000 g, the hardness, springiness, adhesiveness and cohesiveness reached their optimal values, most closely resembling those of the fresh sample. Higher values of hardness, springiness, and cohesiveness after thawing indicated tighter muscle structure, better formability, and greater processing stability, which were beneficial for subsequent processing operations. In addition, lower adhesiveness indicated improved taste and better flavor retention. These changes in texture parameters were closely correlated with thawing loss. As the loading weight increased from 500 g to 2500 g, the thawing loss first decreased and then enhanced. A lower thawing loss reflected that less moisture and less soluble protein were lost during the thawing process, which contributed to better preservation of muscle tissue structure, resulting in a texture more similar to that of the fresh sample, and retaining better flavor. Therefore, at a loading weight of 1000 g, the texture parameters of fish samples after thawing were optimal, yielding large yellow croakers with firm flesh, stable morphology and suitability for further processing.

#### 4.1.7. Specific Energy Consumption (SEC)

**Figure 6:**
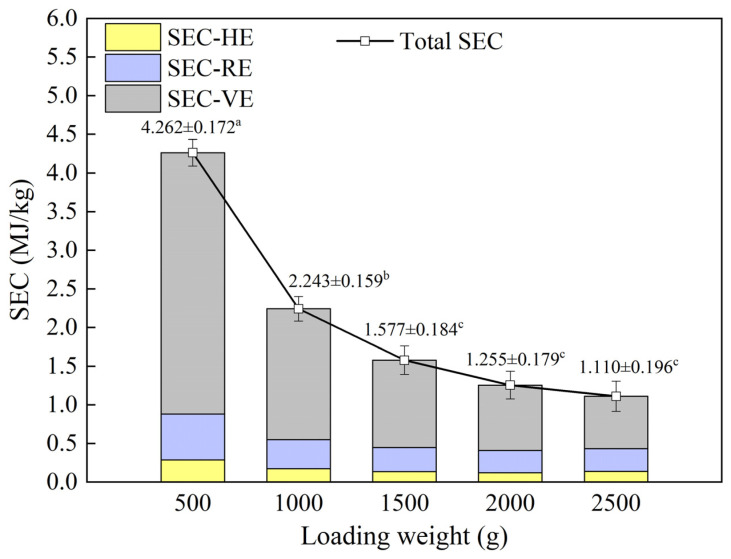
Variations in SEC under different loading weight conditions; within the same figure, different superscripted letters indicate significant differences (*p* < 0.05).

The SEC is the main metric for assessing the energy efficiency of the VSRT system. A lower SEC corresponds to higher energy utilization efficiency. The change in SEC with various loading weight conditions is presented in Figure 6.

As illustrated in Figure 6, when the loading weights were 500, 1000, 1500, 2000, and 2500 g, the total SEC values were 4.262, 2.243, 1.577, 1.255, and 1.110 MJ/kg, respectively. As loading weight increased, the total SEC exhibited a consistent decreasing trend. Similarly, the SEC-VE decreased with increasing loading weight, which could be explained by the fixed energy consumption of vacuum equipment under constant sublimation time, because the correlation between these two variables was positive. On the other hand, both SEC-HE and SEC-RE decreased initially and then enhanced, reaching their minimum values at a loading weight of 2000 g (SEC-HE: 0.121 MJ/kg; SEC-RE: 0.288 MJ/kg). The trends occurred because the operation time of both heating plates (throughout the thawing process) and rehydration equipment (during the rehydration stage) varied dynamically with loading weight. These energy consumptions were collectively termed dynamic energy consumption, and the corresponding SEC was termed dynamic SEC. Under constant radiation power and rehydration temperature, the dynamic energy consumption increased with prolonged thawing time. The dynamic SEC and *STT* were defined as the ratios of dynamic energy consumption and thawing time to loading weight, respectively, so their trends were consistent. Although there was a minimum value in dynamic SEC, the total SEC continued to decline with increasing loading weight, primarily because the SEC-VE accounted for the largest proportion of the total SEC (60.96~79.39%). When the loading weight was 2500 g, the total SEC was the lowest, at only 1.110 MJ/kg. However, as analyzed in the previous section, under this loading weight condition, thawing efficiency, thawing uniformity and thawing effect were all the worst. Therefore, a loading weight of 2500 g was not suitable for batch thawing with priority given to thawing efficiency or effect, but it could be used for thawing large quantities of frozen food with moderate quality requirements. This loading weight condition could significantly save energy consumption and minimize operating costs to the greatest extent.

#### 4.1.8. Comprehensive Evaluation with Different Loading Weights

**Figure 7:**
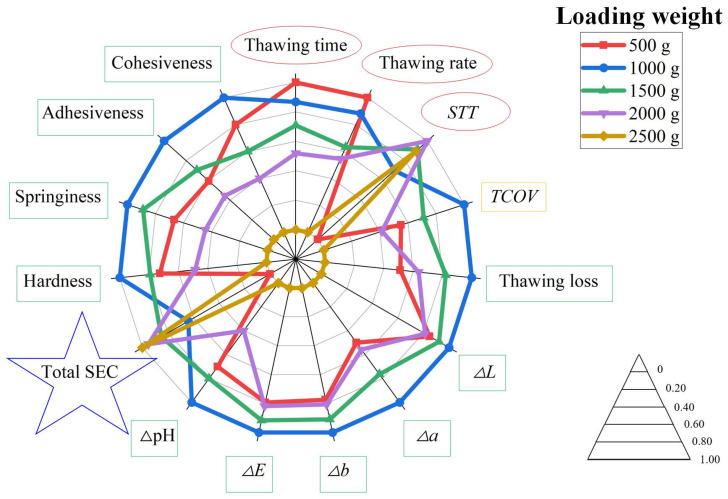
Comprehensive radar chart under different loading weight conditions; red circle: parameters about thawing efficiency; yellow rectangle: parameters about thawing uniformity; green rectangle: parameters about thawing effect; blue star: parameters about energy consumption; Note: larger area enclosed by each operation condition curve means better performance.

To intuitively evaluate the effects of different loading weights on VSRT, the evaluation indexes were normalized and illustrated in a comprehensive radar chart (Figure 7). To facilitate comprehension and enhance the applicability of the results, the loading weight was converted to loading ratio (i.e., the ratio of loading weight to vacuum chamber volume, kg/m^3^).

As presented in Figure 7, based on all evaluation indexes, the optimal loading weight was determined to be 1000 g (i.e., loading ratio of 9.06 kg/m^3^). This condition could achieve the best quality parameters, higher thawing efficiency and lower total SEC. Therefore, at this loading weight condition, both high thawing efficiency and energy conservation could be achieved in VSRT. Under this operation condition, the VSRT system established a PW_F-F_ that facilitated uniform and rapid diffusion of water vapor. With an ice crystal sublimation ratio of 5.05%, efficient and uniform condensation heat transfer between water vapor and fish samples was achieved, which could significantly reduce thawing time and improving thawing uniformity. Thus, the thawing loss was minimized, while the highest moisture content was maintained. Higher moisture content was beneficial for preserving cellular fullness degree and pigment stability, giving fish samples brighter gloss and effectively maintaining textural properties (such as hardness and springiness). This phenomenon resulted in firm texture and stable morphology, which facilitated subsequent processing. Moreover, lower thawing loss indicated reduced nutrient loss, which could prevent excessive decomposition of nitrogen-containing compounds, and thus inhibiting abnormal pH rise. Moreover, a loading weight of 2000 g (i.e., loading ratio of 18.12 kg/m^3^) was established as the maximum for rapid thawing. Beyond this limit, the *STT* increased, and the quality of fish samples further declined. However, for ultra-large-scale thawing industries, the energy consumption required was enormous. If the extent of quality degradation was within an acceptable range, the maximum loading weight of 2500 g (i.e., a loading rate of 22.65 kg/m^3^) could be prioritized as an energy-saving solution due to its lowest total SEC (1.110 MJ/kg), thereby enabling significant electricity savings.

### 4.2. Influence of Different Placement Forms on VSRT

**Figure 8:**
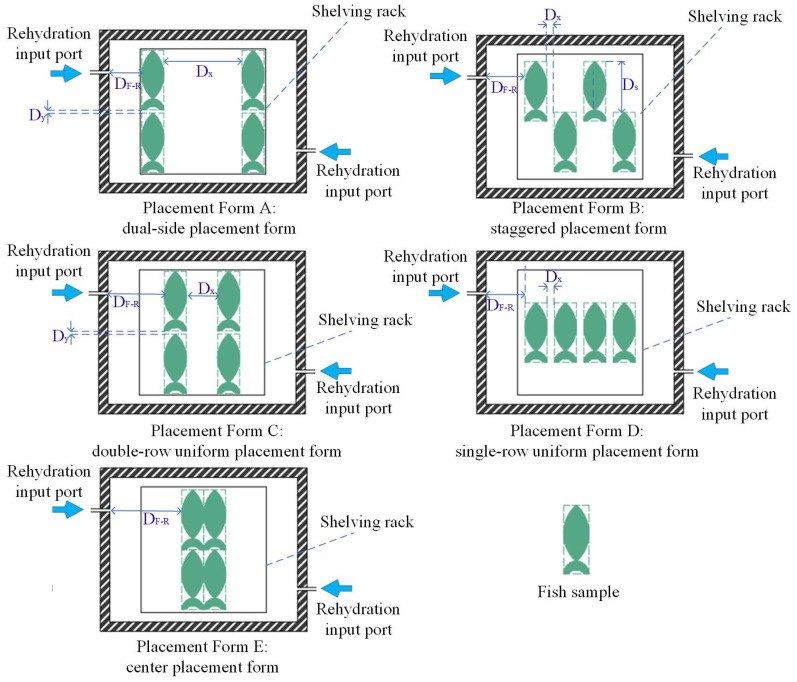
Researched placement forms (top-view cross-section); D_S_ is the staggered distance between adjacent fish samples.

To explore the influence of different placement forms on VSRT, five placement forms were analyzed at the optimal loading weight of 1000 g (as shown in Figure 8 and Table 6).

#### 4.2.1. Influence of Different Placement Forms on Thawing Efficiency and Thawing Uniformity

The alterations in placement form reconstruct the PW_F-F_s and modify the relative positions among fish samples and rehydration input ports, which affected the water vapor diffusion, ultimately influencing both thawing efficiency and thawing uniformity. The changes in thawing efficiency and thawing uniformity with various placement forms are illustrated in Table 7.

As presented in Table 7, both minimal thawing time (91.08 min) and fastest thawing rate (3.30 cm/h) were obtained by Placement Form A. Compared with Placement Form E (the worst), the thawing time declined by 8.07% and the thawing rate enhanced by 11.11%. Meanwhile, Placement Form B yielded the best thawing uniformity with a *TCOV* of 3.78%, a reduction of 46.61% compared to that of Placement Form E. The reasons for these results were as follows:

(1) Placement Form A: The fish samples were positioned closest to the rehydration input ports, allowing water vapor to diffuse most rapidly to fish samples. Therefore, the condensation efficiency was enhanced, leading to the highest thawing efficiency. However, the excessively wide PW_F-F_s were not conducive to the uniform diffusion of water vapor, which declined the thawing uniformity.
(2) Placement Form B: The fish samples were positioned closer to the rehydration input ports, and the widths of PW_F-F_s were moderate. This placement form not only facilitated rapid vapor diffusion to fish samples, but also supported the most uniform condensation heat exchange. Thus, the thawing efficiency was the higher and the thawing uniformity was optimal.
(3) Placement Forms C and D: The fish samples were positioned farther from the rehydration input ports, which slowed the water vapor diffusion to fish samples, resulting in extended thawing time. Additionally, the continuous arrangement of fish samples formed barriers that caused uneven diffusion of water vapor, which declined the thawing uniformity. The double-row uniform placement (Placement Form C) mitigated barriers from outer samples, resulting in better thawing efficiency and improved thawing uniformity compared to those of Placement Form D.
(4) Placement Form E: The fish samples were positioned the farthest from the rehydration input ports and the PW_F-F_s were too narrow, which collectively resulted in the poorest thawing efficiency and thawing uniformity.

#### 4.2.2. Influence of Different Placement Forms on Thawing Effect and Total SEC

Alterations in placement form can alter the diffusion characteristics and condensation efficiency of water vapor, thereby influencing both thawing effect and total SEC. The changes in thawing effect and total SEC with various placement forms are presented in Table 8.

As illustrated in Table 8, the optimal thawing effect was achieved by Placement Form B, reflecting in the lowest thawing loss (0.98%), the highest moisture content (73.59%), the smallest ΔpH (+0.01), the best color and the optimal texture parameters. When the Placement Form A was used, the lowest total SEC (2.171 MJ/kg) was obtained. This phenomenon occurred because the quality of fish samples after thawing was most closely related to the thawing uniformity during the batch thawing process. The adjustment of placement form significantly influenced the diffusion, accumulation and condensation of water vapor, thereby altering the *TCOV*. Placement Form B resulted in the lowest *TCOV* (3.78%), so the optimal thawing effect was obtained. Moreover, since the total SEC was positively correlated with the thawing time, the use of Placement Form A yielded the shortest thawing time (91.08 min). Consequently, the lowest total SEC (2.171 MJ/kg) was achieved with Placement Form A.

#### 4.2.3. Comprehensive Evaluation with Different Placement Forms

**Figure 9:**
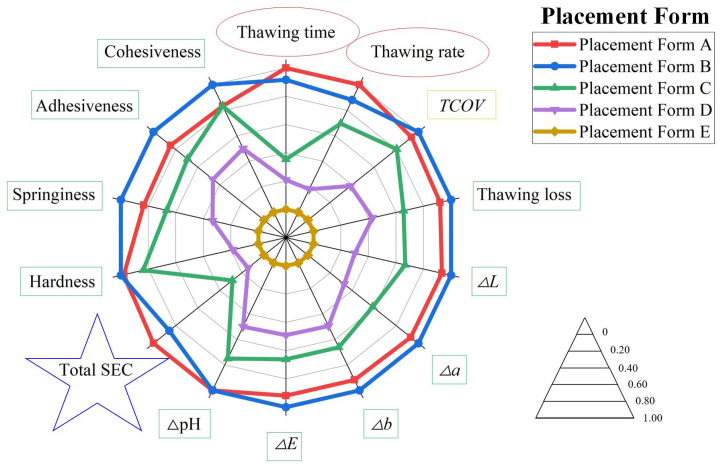
Comprehensive radar chart with different placement forms; red circle: parameters about thawing efficiency; yellow rectangle: parameters about thawing uniformity; green rectangle: parameters about thawing effect; blue star: parameters about energy consumption; note: larger area enclosed by each operation condition curve means better performance.

To intuitively assess the effects of different placement forms on VSRT, the evaluation indexes were normalized and presented in a comprehensive radar chart (Figure 9).

As illustrated in Figure 9, the consideration of all evaluation indexes indicated that Placement Form B yielded the best thawing performance. Similarly to the analysis in Section 4.1.8, Placement Form B (staggered placement form) maintained an appropriate PW_F-F_, facilitating the rapid and uniform diffusion of water vapor and enabling efficient and uniform condensation heat transfer with fish samples. Meanwhile, closer positions of fish samples to rehydration ports further enhanced thawing efficiency, thereby minimizing the thawing loss to the lowest extent and achieving the highest moisture content. Therefore, the quality of fish samples was significantly improved in terms of color, pH, and texture. Additionally, rapid thawing substantially reduced the total SEC. Based on this trend, under Placement Form B, the lowest thawing loss, optimal color, smallest ΔpH and superior texture parameters were obtained. Moreover, higher thawing efficiency and lower total SEC were achieved by Placement Form B, which resulted in a VSRT process that was both highly efficient and energy-saving.

### 4.3. Comparison of Different Thawing Methods

The fundamental principles underlying different thawing methods could significantly affect the thawing efficiency, uniformity, effect and energy consumption. To assess the performance of different batch thawing methods, VSRT (Operation Condition 1: loading weight of 1000 g and Placement Form B) and VSRT (Operation Condition 2: loading weight of 2000 g and Placement Form C) were, respectively, compared with AT (Operation Condition 1; as mentioned above), AT (Operation Condition 2; as mentioned above), VST (Operation Condition 1: as above-mentioned) and VST (Operation Condition 2; as mentioned above). The performance of different thawing methods in batch thawing is summarized in Table 9.

As shown in Table 9, VSRT had the advantages of higher thawing efficiency, better thawing uniformity, lower energy consumption, and improved thawing effect in comparison with AT and VST. The reasons could be attributed to the following. Firstly, the ice crystal sublimation during the sublimation stage created a lot of tiny pores within fish samples, which promoted more uniform and efficient condensation heat exchange during the rehydration stage. Due to the limited thermal conductivity and poor fluidity of air, the heat transfer during AT was inefficient and non-uniform. Therefore, under Operation Conditions 1, compared with AT and VST, the thawing time required for VSRT was shortened by 65.59% and 10.27%, respectively, while the *TCOV* was reduced by 59.39% and 40.28%, respectively. Under Operation Conditions 2, compared with AT and VST, the thawing time required for VSRT was shortened by 49.98% and 10.60%, respectively, while the *TCOV* was reduced by 43.19% and 27.68%, respectively. Next, the quality of fish samples after batch thawing was most closely related to the thawing uniformity. Thus, the fish samples thawed by VSRT exhibited lower thawing loss, higher moisture content, smaller ΔpH, better color, firmer texture, improved formability, and greater processing stability. Finally, the total SEC increased with prolonged thawing time. Therefore, the total SEC required for VSRT was significantly reduced due to the faster thawing rate. Compared with VST, the total SECs required for VSRT were reduced by 6.984 MJ/kg and 5.538 MJ/kg, respectively (i.e., 76.17% and 81.53%). There was no energy consumption in AT, but the thawing effect of AT was the poorest.

## 5. Conclusions

To evaluate the performance of batch thawing large yellow croakers using vacuum sublimation–rehydration thawing, the influence of different loading weights and placement forms on thawing efficiency, thawing uniformity, thawing effect and SEC are investigated in this study. The main conclusions are as follows:

(1) The increase in loading weight resulted in a reduced ice crystal sublimation ratio and the alteration of pathways between adjacent fish samples, which was a key factor influencing vacuum sublimation–rehydration thawing. Based on the experimental system and operation conditions (with constant sublimation time of 20 min, constant D_H-F_ of 80 mm, constant rehydration temperature of 35 °C, and constant heating plate temperature of 35 °C) in this paper, a loading weight of 1000 g (i.e., a loading ratio of 9.06 kg/m^3^) was determined to be optimal, resulting in the water vapor distribution that was most suitable for the operation conditions. This loading weight promoted efficient and uniform condensation heat exchange, which led to the highest thawing uniformity (lowest *TCOV* of 4.22%) and the best thawing effect (lowest thawing loss of 1.24%, highest moisture content of 73.33%, minimum ΔpH of +0.02, optimal color, and superior texture parameters). In addition, the thawing efficiency was higher and the total SEC was lower (2.243 MJ/kg) at a loading weight of 1000 g. The loading weight of 2000 g (i.e., the loading ratio of 18.12 kg/m^3^) was determined to be the maximum for rapid thawing, achieving the shortest specific thawing time (67.83 min/kg), which represented the limiting loading weight for rapid thawing. At a loading weight of 2500 g (i.e., a loading density of 22.65 kg/m^3^), although the lowest total specific energy consumption was achieved (1.110 MJ/kg), it performed relatively the worst in terms of thawing efficiency, thawing uniformity and thawing efficiency. For ultra-large-scale industrial thawing, the required energy consumption was extremely high. If appropriately reducing the quality of thawed food could still meet market demands, the loading density could be increased to 22.65 kg/m^3^ to ensure significant electricity savings.
(2) Different placement forms altered the diffusion path of water vapor, leading to variations in water vapor condensation efficiency, which also significantly influencing vacuum sublimation–rehydration thawing. Applying staggered placement forms was able to provide moderate widths of pathways between adjacent fish samples, and maintain closer distances among the large yellow croakers and rehydration input ports, which promoted efficient and uniform condensation heat exchange between water vapor and fish samples. Thus, the best thawing uniformity (minimum *TCOV* of 3.78%) and the optimal thawing effect (the lowest thawing loss of 0.98%; highest moisture content of 73.59%; minimum ΔpH of +0.01; optimal color; superior texture parameters) were achieved. Moreover, the staggered placement form also resulted in higher thawing efficiency and lower total SEC (2.185 MJ/kg).
(3) Compared with air thawing and vacuum steam thawing, the key advantage of vacuum sublimation–rehydration thawing lay in the formation of tiny pores within frozen food through ice crystal sublimation. This process promoted more uniform and efficient condensation heat exchange between water vapor and large yellow croakers, which improved thawing efficiency and thawing uniformity and obtained optimal thawing loss, pH, color and texture parameters. Additionally, compared with vacuum steam thawing, the total SEC of vacuum sublimation–rehydration thawing was reduced by 76.17~81.53%.
(4) Based on the experimental system and operation conditions in this paper, the recommended loading weight for a high-quality food processing scenario was 1000 g (i.e., loading ratio of 9.06 kg/m^3^) combined with a staggered placement form. This combination could quickly obtain the optimal quality of large yellow croakers and further reduce energy consumption. For scenarios prioritizing higher production capacity, the loading weight could be increased to the maximum of 2000 g (i.e., loading ratio of 18.12 kg/m^3^). However, it must be ensured that the resulting decline in evaluation indexes, such as thawing uniformity, remain within an acceptable range.

In summary, vacuum sublimation–rehydration thawing provides a novel approach for the batch thawing of frozen food that balances quality, efficiency, and energy consumption. The core advantage of this thawing method lies in utilizing ice sublimation to create numerous tiny pores within frozen food, significantly enhancing the uniformity and efficiency of heat and mass transfer, thereby better preserving product quality during thawing. This thawing method can be flexibly adapted to different industrial production requirements by adjusting loading weight and placement form. First, in scenarios with high food quality requirements, a lower loading ratio and optimized placement form can be adopted to achieve the best overall product quality. Second, in scenarios where both production quality and energy efficiency are prioritized, the loading ratio can be moderately increased to reduce energy consumption while ensuring product quality, offering a flexible solution for large-scale production. Third, for ultra-large-scale batch thawing tasks, if the product quality requirement is moderate, the loading ratio can be further maximized to significantly reduce electricity consumption and operational costs, while still ensuring that product quality meets relevant standards. Compared with other thawing methods, vacuum sublimation–rehydration thawing not only performs excellently in quality metrics, but also significantly reduces the total specific energy consumption, which can demonstrate outstanding energy-saving and economic advantages. Therefore, this thawing method is expected to become a key technology for the transition of the frozen food industry towards high efficiency, improved quality, and low carbon emissions. It is particularly suitable for frozen food such as aquatic products, which demands a high thawing effect or large-scale processing, and it has broad prospects for industrial application.

In the future, the research can focus on the following key directions. First, research should be conducted on optimizing the internal water vapor distribution and the spatial layout in thawing equipment. By improving the vacuum chamber structure or the flow-guiding design, the loading capacity, thawing efficiency and thawing uniformity can be further enhanced, ensuring consistent production of high-quality thawed food. Second, an intelligent control system capable of real-time monitoring of thawing uniformity and energy consumption should be developed to dynamically adjust the loading layout and process parameters based on the frozen food shape and production capacity requirements, thereby enhancing production adaptability and energy utilization efficiency. Finally, a standardized loading scheme database for different frozen food can be established, clearly defining the optimal loading ratio range and corresponding placement norms to achieve standardized control of food quality and energy efficiency.

## Figures and Tables

**Table 1:** Instruments and equipment.

Instruments/Equipment	Manufacturers/Brands	Parameters	Accuracy
Roots pump	Jiangyin Tiantian Vacuum Equipment Manufacturing Co., Ltd. (ZJP-70), Jiangyin, China	Ultimate pressure: 0.05 Pa	±0.01 Pa
Rotary vane pump	Leybold SOGEVAC (SV40B), Dresden, Germany	Ultimate pressure: 150 Pa	±1 Pa
Water vapor generator	Beijing Yong Guangming Medical Instruments (SXTW), Beijing, China	0~399 °C	±1 °C
Omron thermostat	Omron (E5CC-800), Tokyo, Japan	20~180 °C	±0.5 °C
Texture analyzer	Stable Micro System (TA-XT2i), Surrey, UK	Test distance: 0.1~300 mm Test stress: 0~50 kg Test speed: 0.01~40 mm/s	±0.001 mm ±0.1 g
Power meter	UNI-T (UT230E), Dongguan, China	0.001~9999 kWh	±1% Rdg
Pressure sensor	GE-Druck (UNIK5000), Billerica, MA, USA	0~100 kPa	±0.04% FS
Mass sensor	Jinnuo (JLBS-M2), Beijing, China	0~3000 g	±0.5 g
Temperature sensor	Omega v.8.0.0. (T-type, TT-T-30-SLE), Norwalk, CT, USA	−200~350 °C	±0.5 °C
Colorimeter	Konica Minolta (CR-400), Tokyo, Japan	0.01~160% (reflectivity)	±6% FS
pH meter	Sigma Instruments (PH8180-0-00), Luoyang, China	0.00~14.00 pH	±0.05 pH
Electronic balance	Sartorius (BSA3202S), Göttingen, Germany	0~3.2 kg	±0.01 g

**Table 2:** Size parameters for Figure 3.

Loading Weight	Sample Number	Sample Size	D_x_	D_y_	D_F-R_
500 g	2	70 mm × 190 mm	100 mm	-	180 mm
1000 g	4	70 mm × 190 mm	100 mm	10 mm	180 mm
1500 g	6	70 mm × 190 mm	47.5 mm	10 mm	147.5 mm
2000 g	8	70 mm × 190 mm	24 mm	10 mm	124 mm
2500 g	10	70 mm × 190 mm	8 mm	10 mm	109 mm

**Table 3:** Total mass of fish samples at various stages.

Before Thawing	Sublimation Stage Completed			Rehydration Stage Completed		
*M*_0_ (g)	*M_i_* (g)	*M*_0_ − *M_i_* (g)	Ice Crystal Sublimation Ratio (*M*_0_ − *M_i_*)/*M*_0_	*M*_1_ (g)	*M*_1_ − *M_i_* (g)	Water Replenishment Ratio (*M*_1_ − *M_i_*)/*M*_0_
500	472.92	27.08	5.42%	491.66	18.74	3.75%
1000	949.48	50.52	5.05%	987.57	38.09	3.81%
1500	1443.54	56.46	3.76%	1479.03	35.49	2.37%
2000	1935.76	64.24	3.21%	1968.82	33.06	1.65%
2500	2435.50	64.50	2.58%	2447.09	11.59	0.46%

Note: *M_i_* represents the total mass of fish samples after ice crystal sublimation; *M*_1_ represents the total mass of fish samples after the rehydration stage.

**Table 4:** Thawing loss and color parameters and pH.

Loading Weight/State	Thawing Loss (%)	Moisture Content (%)	*L*	*a*	*b*	Δ*E*	pH
Fresh sample		74.57 ± 0.05 ^a^	77.20 ± 0.11 ^a^	4.49 ± 0.09 ^a^	26.85 ± 0.10 ^b^	-	6.51 ± 0.01 ^b^
500 g	1.67 ± 0.06 ^a^	72.90 ± 0.06 ^b^	76.83 ± 0.09 ^a^	3.82 ± 0.10 ^b^	31.13 ± 0.11 ^a^	4.35 ± 0.17 ^b^	6.56 ± 0.01 ^ab^
1000 g	1.24 ± 0.04 ^c^	73.33 ± 0.04 ^b^	77.08 ± 0.12 ^a^	3.99 ± 0.08 ^b^	30.78 ± 0.09 ^a^	3.97 ± 0.17 ^c^	6.53 ± 0.01 ^b^
1500 g	1.40 ± 0.05 ^b^	73.17 ± 0.05 ^b^	76.95 ± 0.08 ^a^	3.91 ± 0.10 ^b^	30.92 ± 0.09 ^a^	4.12 ± 0.16 ^b^	6.55 ± 0.01 ^ab^
2000 g	1.56 ± 0.05 ^b^	73.01 ± 0.05 ^b^	76.77 ± 0.07 ^a^	3.84 ± 0.10 ^b^	31.08 ± 0.10 ^a^	4.30 ± 0.16 ^b^	6.59 ± 0.01 ^ab^
2500 g	2.12 ± 0.06 ^a^	72.44 ± 0.06 ^b^	75.43 ± 0.10 ^b^	3.65 ± 0.08 ^c^	32.33 ± 0.11 ^a^	5.82 ± 0.17 ^a^	6.63 ± 0.01 ^a^

Note: Within the same column, different superscripts indicate significant differences (*p* < 0.05).

**Table 5:** Texture parameters of fish samples.

Loading Weight/State	Hardness (gf)	Springiness	Adhesiveness (g·s)	Cohesiveness
Fresh sample	2416.8 ± 151.1 ^a^	0.73 ± 0.02 ^a^	6.84 ± 0.38 ^b^	0.36 ± 0.03 ^a^
500 g	2143.2 ± 165.8 ^ab^	0.67 ± 0.04 ^abc^	7.89 ± 0.30 ^a^	0.30 ± 0.04 ^ab^
1000 g	2290.6 ± 154.7 ^ab^	0.70 ± 0.03 ^ab^	7.78 ± 0.24 ^a^	0.31 ± 0.02 ^ab^
1500 g	2178.9 ± 135.2 ^ab^	0.69 ± 0.02 ^ab^	7.86 ± 0.21 ^a^	0.29 ± 0.02 ^ab^
2000 g	2013.8 ± 152.0 ^b^	0.65 ± 0.03 ^bc^	7.93 ± 0.29 ^a^	0.28 ± 0.05 ^b^
2500 g	1752.1 ± 132.9 ^c^	0.61 ± 0.04 ^c^	8.05 ± 0.36 ^a^	0.26 ± 0.05 ^b^

Note: Within the same column, different superscripts indicate significant differences (*p* < 0.05).

**Table 6:** Size parameters about Figure 8.

Placement Form	Sample Size	D_x_	D_y_	D_F-R_	D_S_
A	70 mm × 190 mm	250 mm	10 mm	105 mm	-
B	70 mm × 190 mm	24 mm	-	124 mm	162 mm
C	70 mm × 190 mm	100 mm	10 mm	180 mm	-
D	70 mm × 190 mm	24 mm	-	124 mm	-
E	70 mm × 190 mm	0	0	230 mm	-

**Table 7:** Thawing uniformity and thawing efficiency with various placement forms.

Placement Form	Thawing Time (min)	Thawing Rate (cm/h)	*TCOV* (%)
A	91.08 ± 1.17 ^b^	3.30 ± 0.02 ^a^	3.98 ± 0.26 ^c^
B	91.75 ± 1.22 ^b^	3.26 ± 0.01 ^a^	3.78 ± 0.23 ^c^
C	96.25 ± 1.19 ^a^	3.20 ± 0.01 ^b^	4.42 ± 0.25 ^bc^
D	97.42 ± 1.18 ^a^	3.03 ± 0.02 ^c^	5.80 ± 0.27 ^b^
E	99.08 ± 1.24 ^a^	2.97 ± 0.02 ^c^	7.08 ± 0.28 ^a^

Note: Within the same column, different superscripted letters indicate significant differences (*p* < 0.05).

**Table 8:** Thawing effect and total SEC under different placement forms.

Placement Form/State	Thawing Loss	pH	*L*	*a*	*b*	Δ*E*
Fresh sample	-	6.51 ± 0.01 ^a^	77.20 ± 0.11 ^a^	4.49 ± 0.09 ^a^	26.85 ± 0.10 ^b^	-
A	1.04 ± 0.04 ^c^	6.52 ± 0.01 ^a^	77.16 ± 0.11 ^a^	4.19 ± 0.08 ^ab^	30.06 ± 0.08 ^a^	3.23 ± 0.16 ^c^
B	0.98 ± 0.04 ^c^	6.52 ± 0.01 ^a^	77.18 ± 0.11 ^a^	4.23 ± 0.09 ^ab^	29.83 ± 0.08 ^a^	3.00 ± 0.16 ^c^
C	1.24 ± 0.04 ^b^	6.53 ± 0.01 ^a^	77.08 ± 0.12 ^a^	3.99 ± 0.08 ^b^	30.78 ± 0.09 ^a^	3.97 ± 0.17 ^b^
D	1.42 ± 0.05 ^b^	6.54 ± 0.01 ^a^	76.97 ± 0.13 ^a^	3.84 ± 0.10 ^c^	31.25 ± 0.11 ^a^	4.46 ± 0.20 ^b^
E	1.75 ± 0.06 ^a^	6.56 ± 0.01 ^a^	76.88 ± 0.15 ^a^	3.65 ± 0.10 ^d^	32.64 ± 0.13 ^a^	5.86 ± 0.22 ^a^
Placement form/State	Moisture content (%)	Total SEC (MJ/kg)	Hardness (gf)	Springiness	Cohesiveness	Adhesiveness (g·s)
Fresh sample	74.57 ± 0.05 ^a^	-	2416.8 ± 151.1 ^a^	0.73 ± 0.02 ^a^	0.36 ± 0.03 ^a^	6.84 ± 0.38 ^c^
A	73.53 ± 0.04 ^b^	2.171 ± 0.136 ^a^	2380.3 ± 162.6 ^a^	0.71 ± 0.02 ^ab^	0.31 ± 0.02 ^b^	7.76 ± 0.33 ^b^
B	73.59 ± 0.04 ^b^	2.185 ± 0.127 ^a^	2393.9 ± 155.2 ^a^	0.72 ± 0.02 ^a^	0.32 ± 0.01 ^b^	7.74 ± 0.22 ^b^
C	73.33 ± 0.04 ^b^	2.243 ± 0.159 ^a^	2290.6 ± 154.7 ^a^	0.70 ± 0.03 ^ab^	0.31 ± 0.02 ^b^	7.78 ± 0.24 ^b^
D	73.15 ± 0.05 ^b^	2.257 ± 0.167 ^a^	1875.7 ± 156.7 ^b^	0.68 ± 0.03 ^ab^	0.29 ± 0.03 ^b^	7.81 ± 0.24 ^a^
E	72.82 ± 0.06 ^b^	2.272 ± 0.174 ^a^	1758.9 ± 153.3 ^b^	0.66 ± 0.04 ^b^	0.26 ± 0.04 ^c^	7.87 ± 0.22 ^a^

Note: Within the same column, different superscripted letters indicate significant differences (*p* < 0.05).

**Table 9:** Comparison of different thawing methods (Operation Condition: OC).

Thawing Method/State	Thawing Time (min)	Thawing Rate (cm/h)	*TCOV* (%)	Thawing Loss (%)	pH
Fresh sample	-	-	-	-	6.51 ± 0.01 ^c^
VSRT (OC 1)	91.75 ± 1.22 ^c^	3.26 ± 0.01 ^a^	3.78 ± 0.23 ^c^	0.98 ± 0.04 ^c^	6.52 ± 0.01 ^c^
VSRT (OC 2)	135.67 ± 1.24 ^b^	2.82 ± 0.02 ^b^	9.8 ± 0.39 ^b^	1.56 ± 0.05 ^bc^	6.59 ± 0.01 ^c^
AT (OC 1)	266.67 ± 1.78 ^a^	0.63 ± 0.04 ^c^	9.31 ± 0.49 ^b^	9.03 ± 0.07 ^a^	6.97 ± 0.01 ^a^
AT (OC 2)	271.25 ± 1.93 ^a^	0.57 ± 0.03 ^c^	17.25 ± 0.47 ^a^	9.32 ± 0.08 ^a^	6.99 ± 0.01 ^a^
VST (OC 1)	102.25 ± 1.33 ^c^	2.67 ± 0.02 ^b^	6.33 ± 0.35 ^bc^	2.72 ± 0.06 ^b^	6.64 ± 0.01 ^bc^
VST (OC 2)	151.75 ± 1.38 ^b^	2.51 ± 0.03 ^b^	13.55 ± 0.43 ^ab^	3.38 ± 0.06 ^b^	6.73 ± 0.01 ^b^
Thawing method/State	*L*	*a*	*b*	Δ*E*	Total SEC (MJ/kg)
Fresh sample	77.20 ± 0.11 ^a^	4.49 ± 0.09 ^a^	26.85 ± 0.10 ^c^	-	-
VSRT (OC 1)	77.18 ± 0.11 ^a^	4.23 ± 0.09 ^a^	29.83 ± 0.08 ^bc^	3.00 ± 0.16 ^c^	2.185 ± 0.127 ^b^
VSRT (OC 2)	76.77 ± 0.07 ^a^	3.84 ± 0.10 ^b^	31.08 ± 0.10 ^b^	4.30 ± 0.16 ^bc^	1.255 ± 0.179 ^bc^
AT (OC 1)	67.35 ± 0.11 ^c^	1.83 ± 0.12 ^d^	38.73 ± 0.10 ^a^	15.67 ± 0.19 ^a^	0 ^c^
AT (OC 2)	66.91 ± 0.12 ^c^	1.66 ± 0.10 ^d^	39.43 ± 0.12 ^a^	16.50 ± 0.19 ^a^	0 ^c^
VST (OC 1)	75.78 ± 0.09 ^ab^	3.37 ± 0.11 ^b^	30.89 ± 0.10 ^b^	4.43 ± 0.17 ^bc^	9.169 ± 0.156 ^a^
VST (OC 2)	73.21 ± 0.12 ^b^	3.15 ± 0.08 ^c^	31.75 ± 0.11 ^b^	6.46 ± 0.18 ^b^	6.793 ± 0.183 ^ab^
Thawing method/State	Moisture content (%)	Hardness (gf)	Springiness	Cohesiveness	Adhesiveness (g·s)
Fresh sample	74.57 ± 0.05 ^a^	2416.8 ± 151.1 ^a^	0.73 ± 0.02 ^a^	0.36 ± 0.03 ^a^	6.84 ± 0.38 ^c^
VSRT (OC 1)	73.59 ± 0.04 ^b^	2393.9 ± 155.2 ^a^	0.72 ± 0.02 ^a^	0.32 ± 0.01 ^a^	7.74 ± 0.22 ^ab^
VSRT (OC 2)	73.01 ± 0.05 ^c^	2013.8 ± 152.0 ^bc^	0.65 ± 0.03 ^ab^	0.28 ± 0.05 ^b^	7.93 ± 0.29 ^a^
AT (OC 1)	65.54 ± 0.07 ^e^	1851.4 ± 158.9 ^d^	0.55 ± 0.05 ^bc^	0.23 ± 0.03 ^d^	7.47 ± 0.21 ^d^
AT (OC 2)	65.25 ± 0.08 ^e^	1791.8 ± 161.7 ^d^	0.51 ± 0.04 ^c^	0.22 ± 0.05 ^d^	7.64 ± 0.25 ^b^
VST (OC 1)	71.85 ± 0.06 ^d^	2274.1 ± 160.7 ^b^	0.64 ± 0.03 ^ab^	0.29 ± 0.02 ^b^	7.52 ± 0.31 ^c^
VST (OC 2)	71.19 ± 0.06 ^d^	1955.5 ± 178.1 ^c^	0.61 ± 0.03 ^abc^	0.25 ± 0.06 ^c^	7.71 ± 0.33 ^ab^

Note: Within the same column, different superscripted letters indicate significant differences (*p* < 0.05).

## Data Availability

The original contributions presented in the study are included in the article, further inquiries can be directed to the corresponding author.
